# Phenotype and genotype of hypophosphatasia cases in Saudi Arabia: multi-center case cohort

**DOI:** 10.3389/fgene.2025.1715818

**Published:** 2026-01-13

**Authors:** Afaf Alsagheir, Ali Mcrabi, Meshari Alquayt, Raghad Alhuthil, Afnan Alawi, Eissa Faqeih, Abrar Turki Alabdullatif, Doua Al Homyani, Amal AlJohany, Mariam AlOtaibi, Magdy Rabea, Hassan AlSayed, Mohamed H. Al-Hamed

**Affiliations:** 1 Pediatric Endocrinology Section, Department of Pediatrics, King Faisal Specialist Hospital & Research Centre, Riyadh, Saudi Arabia; 2 Department of Pediatrics, King Faisal Specialist Hospital & Research Centre, Riyadh, Saudi Arabia; 3 Pediatric Endocrinology Section, Department of Pediatrics, King Fahad Specialist Hospital (Eastern Health Cluster), Dammam, Saudi Arabia; 4 Section of Medical Genetics, Children’s Specialist Hospital, King Fahad Medical City, Riyadh, Saudi Arabia; 5 Pediatric Endocrinology Section, Department of Pediatrics, King Fahad General Hospital-AlHasa, AlHasa, Saudi Arabia; 6 Pediatric Endocrinology Section, Department of Pediatrics, Children’s Hospital Taif, Taif, Saudi Arabia; 7 Pediatric Endocrinology Section, Department of Pediatrics, Maternity and Children Hospital, Madinah, Saudi Arabia; 8 Medical Affairs Department, AstraZeneca GCC, Riyadh, Saudi Arabia; 9 Centre for Genomic Medicine, KFSH&RC, Riyadh, Saudi Arabia

**Keywords:** ALPL gene, asfotase alfa, enzyme replacement therapy, hypophosphatasia, rare hereditary disorder

## Abstract

**Introduction:**

Hypophosphatasia (HPP) is a rare inherited metabolic disease caused by mutations in the *ALPL* gene. The disease is heterogeneous, complicating its diagnosis and delaying optimal management, leading to severe or lethal outcomes such as failure to thrive, fragility fractures, bone deformities, delayed motor development, respiratory failure, seizures, and premature death. However, no epidemiological studies on the incidence of HPP in Saudi Arabia have been identified until now. Therefore, the study aimed to describe the phenotype and genotype of Saudi patients with HPP.

**Methods:**

This retrospective multicenter case series included six centers in Saudi Arabia. Paediatrics and adult patients with clinically and genetically confirmed HPP were included between January 2014 and May 2024. Demographic and clinical information, including medical history, clinical, biochemical, genetic, and management data, was collected retrospectively from medical records and summarized descriptively. Additionally, whole-exome sequencing or ALPL next-generation sequencing (NGS) was performed. Furthermore, pre- and post-analysis for patients who received asfotase alfa was performed using the Wilcoxon signed-rank test.

**Results:**

The study included 19 HPP cases, of whom 68.4% were male. There were five patients with perinatal onset (26.3%), 13 with infantile onset (68.4%), and one with childhood onset (5.3%) of HPP. About 78.9% of patients indicated a family history of HPP; consanguinity was observed in nearly all parents of cases. Bone deformities were observed in all patients, including skull (78.5%), limb (100%), spinal (49.9%), and dental abnormalities (57.9%). Complications such as craniosynostosis (78.5%), nephrocalcinosis (26.3%), kyphoscoliosis (49.9%), and convulsions (26.3%) were also documented, with 4 (21.05%) deaths. Thirteen (68.4%) of our patients received asfotase alfa. All cases tested positive for *ALPL* variants, with the most common being c.293C>T (p.Ser98Phe) and c.977G>T (p.Gly326Val), both of which were novel and not previously reported.

**Conclusion:**

Our study highlights HPP’s diverse phenotypes and genotypes in Saudi Arabia, revealing distinct *ALPL* mutations. We identified a high prevalence of consanguinity and family histories of HPP. Treatment with asfotase alfa was generally effective and safe.

## Introduction

Hypophosphatasia (HPP) is a rare inherited metabolic bone disease caused by mutations in the *ALPL* gene on chromosome 1p36.12, encoding the tissue-nonspecific isoenzyme of alkaline phosphatase (TNSALP), leading to a reduction in serum alkaline phosphatase (ALP) (Whyte, 2016; Im and Cho, 2025; Dahir and Dunbar, 2025). This enzymatic deficiency leads to the accumulation of several metabolites, including pyridoxal 5′-phosphate (PLP), inorganic pyrophosphate (PPi), and phosphoethanolamine. It was found that the accumulation of PPi results in the inhibition of bone mineralization, while impaired PLP dephosphorylation induces seizures in addition to other neurological consequences (Linglart and Biosse-Duplan, 2016; Colazo et al., 2019; Kishnani et al., 2025).

HPP may be inherited in either an autosomal recessive or dominant manner. About 74% of *ALPL* gene mutations, mainly responsible for HPP, are missense mutations (Villa-Suárez et al., 2021). Notably, life-threatening HPP in infants is usually inherited as autosomal recessive or autosomal dominant mutation with a dominant negative effect, in which the gene product from one allele interferes with homo-dimerization, whereas HPP at other ages could be inherited as autosomal recessive or dominant (Villa-Suárez et al., 2021; Reis and Lazaretti-Castro, 2023).

The clinical manifestations of HPP are heterogeneous, and the most common symptoms involve hypomineralization of bone and/or teeth, premature loss of teeth, failure to thrive, fragility fractures, bone deformities, and delayed motor development, and it might progress leading to pulmonary hypoplasia, seizures, respiratory failure and premature death (Reis and Lazaretti-Castro, 2023). Historically, HPP was classified into five different clinical phenotypes according to the age of onset of disease symptoms and the patient’s age into perinatal, infantile, childhood, and adult (Reis and Lazaretti-Castro, 2023).

Based on epidemiological studies, the birth prevalence of perinatal/infantile HPP is about 1 in 300,000 births in European populations, 1 in 100,000 births in the Canadian population, and 1 in 300,000–500,000 births in the Japanese population (Linglart and Biosse-Duplan, 2016; Reis and Lazaretti-Castro, 2023; Whyte, 2013; Greenberg et al., 1993; Mornet et al., 2011; Leung et al., 2013; Taketani et al., 2014). At the same time, the prevalence of the HPP could be more frequent and has been estimated at 1/6,000 (Mornet, 2015). However, this prevalence might be overestimated due to the reliance on *ALPL* mutation analysis, which does not account for the signs and symptoms of the disease, as well as variable penetrance and expressivity. Thus, the overall prevalence of HPP remains unclear. Additionally, no epidemiological studies on the incidence of HPP in Saudi Arabia have been identified until now.

Furthermore, the wide range of *ALPL* mutations leads to numerous combinations of compound heterozygous variants, which increases the variability of HPP clinical expression and complicates the diagnosis of HPP for healthcare professionals.

To address the limited data on HPP in the Saudi Arabian population, this study aimed to provide a detailed description of the phenotypic and genotypic profiles of Saudi patients with HPP, offering critical insights to enhance clinical management and inform future research in this population.

## Methods

### Study design and patients

This retrospective multicentre study involved six centers in Saudi Arabia: King Faisal Specialist Hospital and Research Center (KFSHRC) in Riyadh, King Fahad Medical City in Riyadh, King Fahad Hospital in Al-Ahsa, Taif Children’s Hospital, Maternity and Children’s Hospital (MMCH) in Al-Madinah, and King Fahad Specialist Hospital. The study included pediatric and adult patients clinically or genetically confirmed to have HPP between January 2014 and May 2024. The age at diagnosis was categorized into three groups: perinatal, infantile (from 1 to 6 months of age), and childhood (from 7 months up to 18 years). The diagnosis of HPP was established using standardized clinical, radiologic, and biochemical findings (including persistently low ALP), regardless of whether ALPL genetic testing was performed (Rush et al., 2024). The study was designed according to the Declaration of Helsinki, and ethical approval was obtained from the Office of Research Affairs in King Faisal Specialist Hospital and Research Centre (Reference number: 2241191).

### Data Collection

The study gathered the following data from medical records: demographics, medical history, clinical presentations, genetic findings, growth data, management details, biochemical data, and complications. The findings of the following biochemical tests were extracted, including serum ALP, calcium, phosphate, and vitamin D. Age-specific reference ranges were applied for ALP: 130–260 IU/L for children aged 3 to <10 years, 130–340 IU/L for those aged 10 to <14 years, 30–180 IU/L for adolescents aged 14–18 years, and 30–130 IU/L for adults (>18 years). Vitamin D deficiency was defined as a serum 25(OH)D concentration <50 nmol/L. Reference ranges for serum phosphate were 0.90–1.80 mmol/L in children ≤16 years and 0.80–1.50 mmol/L in adults >16 years. The normal reference range for serum calcium was 2.10–2.54 mmol/L.

### Genetic analysis and ALPL gene sequencing

Genetic testing was performed as part of routine clinical practice. After obtaining patient consent, DNA was extracted from peripheral blood cells using the Gentra Systems PUREGENE DNA Isolation kit (Qiagen, Valencia, California, United States). All cases underwent Whole-exome sequencing (WES), performed according to a previously published protocol. Briefly, genomic DNA libraries were prepared using the Agilent SureSelect All Exons V6 (50 Mb) capture kit and sequenced on the Illumina HiSeq 2,500 platform, achieving an average target coverage of 80×. Reads were aligned to the human reference genome (NCBI build 37.1; UCSC hg19). Data analysis was performed using QIAGEN Clinical Insight (QCI) Interpret, which includes copy-number variation (CNV) detection, along with an in-house variant interpretation pipeline. The in-house resources include databases of known pathogenic variants found in the Saudi Arabian population and an aggregated variant dataset created from samples processed at the Center for Genomic Medicine (CGM-DB). Variant classification was based on the American College of Medical Genetics and Genomics (ACMG) guidelines (Richards et al., 2015; Al-Hamed et al., 2022).

### Statistical analysis

Data analysis was performed using STATA version 18. Continuous data was reported using mean ± standard deviation (SD) and median and interquartile range [IQR]. Categorical data were described with frequencies and percentages. Furthermore, pre- and post-analysis for patients who received asfotase alfa were performed using the Wilcoxon signed-rank test to assess the change in height and laboratory data (serum ALP, calcium, phosphate, and vitamin D). P < 0.05 was considered statistically significant.

## Results

### Study population

**FIGURE 1:**
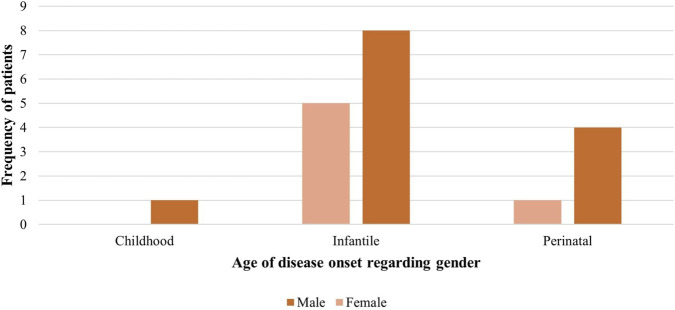
Frequency of patients regarding their gender and disease onset Stage.

The study population comprised 19 HPP cases from 6 medical centres in Saudi Arabia, and 68.4% of them were males. The median age of the subjects was 9 years old [IQR: 0.3, 15], and the median age of diagnosis was 5.5 months [IQR: 0.8, 16]. Our cohort consisted of five perinatal (26.3%), 13 infantile (68.4%), and one childhood (5.3%) onset HPP patient (Table 1; Figure 1). There were three perinatal (23%), nine infantile (69%), and one childhood (7.7%) onset HPP patients treated with asfotase alfa. Approximately 78.9% of patients reported a family history of HPP, and consanguinity was observed among the parents of almost all patients (94.7%). Most patients treated with asfotase alfa (92%) had a family history of HPP, while only half of those who were not treated with asfotase alfa had a family history of the disease (3 patients; 50%). Moreover, all cases tested positive for *ALPL* variants. Regarding the anthropometric measures, the median mid-parental height was 169 cm [IQR: 158, 170.5], and the median baseline SDS height was −3.5 [IQR: −5.9, −2.4] (Table 1; Figure 1).

**TABLE 1:** Demographic data of hypophosphatasia patients (n = 19).

Variables	Total (N = 19)	Non-treated (n = 6)	Treated (n = 13)	p-value (exploratory)
Gender				
Male	13 (68.4)	4 (67%)	9 (69%)	<0.001*
Female	6 (31.6)	2 (33%)	4 (31%)	
Current age (years)	9.35 ± 8.18	9.14 ± 10.12	9.45 ± 7.60	0.8231
	9 [0.3,15]	7 [0, 19]	9 [2, 15]	
Age at diagnosis (months)	18.61 ± 30.53	4.06 ± 4.46	24.20 ± 34.53	0.4261
	5.5 [0.8,16]	2.65 [0, 11]	7 [0, 84]	
Age of disease onset		​	​	<0.001*
Perinatal	5 (26.3)	2 (33%)	3 (23%)	​
Infantile	13 (68.4)	4 (67%)	9 (69%)	​
Childhood	1 (5.3)	0 (0%)	1 (7.7%)	​
Positive family history		​	​	<0.001*
Yes	15 (78.9)	3 (50%)	12 (92%)	​
No	4 (21.1)	3 (50%)	1 (8%)	​
Consanguinity		​	​	<0.001*
Yes	18 (94.7)	5 (83%)	13 (100%)	​
No	1 (5.3)	1 (17%)	0 (0%)	​
Genetic test done	19 (100)	6 (100%)	13 (100%)	NA
Positive for *ALPL Variants*	19 (100)	6 (100%)	13 (100%)	NA
Mid parental height (cm)	169 [158, 170.5]	169 [-]	163.5 [158, 176]	0.843^a^
Baseline height (cm)	70.67 ± 26.6	88.40 ± 48.96	67.17 ± 16.62	0.533^b^
Last visit height (cm)	128.75 [88.20, 140.25]	140.75 [138, 143.5]	123.25 [58, 162]	0.133^a^
Baseline height SDS	−3.99 ± 1.92	−4.0 ± 1.61	−3.76 ± 2.08	0.861^b^
Last visit height SDS	−3.30 ± 1.78	−4.23 ± 0.50	−3.12 ± 1.90	0.448^b^
Growth velocity cm/year	3.83 ± 1.89	2.38 ± 0.72	4.25 ± 1.94	0.240^b^
Follow-up period (years)	6.40 ± 4.67	9.50 ± 4.95	5.43 ± 4.92	0.337^b^

SDS, Standard Deviation Score using CDC, growth chart, * Significant (Fisher-exact test).

^a^ Mann-Whitney test.

^b^ T. test.

^c^ (%), mean ± SD, median [IQR].

### Biochemical and clinical characteristics of the study population

Regarding clinical manifestations, deformities were observed among all patients, including skull (n = 11; 78.5%), limbs (n = 19; 100%), spinal (n = 7; 49.9%), dental (n = 11; 57.9%), and chest (n = 4; 28.6%) deformities. There were seven (36.8%) cases with distinctive facial features, and eight (42.1%) reported a history of fractures, half of which were single fractures. Craniosynostosis (n = 11; 78.5%), nephrocalcinosis (n = 5; 26.3%), of which more than half had grade 2–3 nephrocalcinosis (n = 3; 60%), and kyphoscoliosis (n = 7; 49.9%). Other comorbidities were also reported, most of them were chronic respiratory disease or infection (n = 6; 31.57%), followed by respiratory support need (n = 5; 26.3%), convulsions (n = 5; 26.3%), and speech delay (n = 1; 5.3%) (Table 2).

**TABLE 2:** Clinical features, laboratory findings of patients (n = 19).

Variables	n (%), median [IQR]
Clinical features	
Deformities^a^	
Yes	19 (100)
Types of deformities (n = 19)	
Skull	11 (78.5)
Limbs	19 (100)
Spinal	7 (49.9)
Dental	11 (57.9)
Chest	4 (28.6)
Distinctive facial features	
Yes	7 (36.8)
No	12 (63.2)
Fractures	
Yes	8 (42.1)
No	11 (57.9)
Number of fractures (n = 8)	
1	4 (50)
4	1 (12.5)
Multiple	3 (37.5)
Craniosynostosis	
Yes	11 (78.5)
No	8 (21.5)
Nephrocalcinosis	
Yes	5 (26.3)
No	14 (73.7)
Grade of nephrocalcinosis (n = 5)	
Grade 1	1 (20)
Grade 2	1 (20)
Grade 2–3	3 (60)
Kyphoscoliosis	
Yes	7 (49.9)
No	13 (68.4)
Other comorbidities	
Convulsions	5 (26.3)
Need respiratory support	5 (26.2)
Chronic respiratory disease/infection	6 (31.57)
Speech delay	1 (5.3)
Laboratory tests at baseline	
ALP (U/L) (n = 17)	19 [7, 30]
Ca (mmol/L) (n = 18)	2.51 [2.42, 2.60]
Phosphate (mmol/L) (n = 17)	1.87 [1.51, 2.30]
Vitamin D (nmol/L) (n = 12)	75.69 [23.25, 90.50]
Laboratory tests at the last visit	
ALP (U/L) (n = 15)	1,504 [246, 60,000]
Ca (mmol/L) (n = 15)	2.49 [2.42, 2.56]
Phosphate (mmol/L) (n = 15)	1.93 [1.52, 2.00]
Vitamin D (nmol/L) (n = 12)	46.50 [20.25, 81.00]

^a^ 12 patients had more than one deformity.

All patients showed reduced median serum levels of ALP at baseline, 19 U/L [IQR: 7, 30], which increased significantly during the last visit, 1504 U/L [IQR: 246, 60,000]. Meanwhile, the median calcium, phosphate, and vitamin D levels were stable from baseline to the last visit (2.51 mmol/L [IQR: 2.42, 2.6] vs. 2.49 mmol/L [IQR: 2.42, 2.56]), (1.87 mmol/L [IQR: 1.51, 2.30] vs. 1.93 mmol/L [IQR: 1.52, 2]) and (75.69 nmol/L [IQR: 23.25, 90.50] vs. 46.5 nmol/L [IQR: 20.25, 81]), respectively (Table 2).

### Therapeutic outcomes of asfotase alfa and disease complications

There were six (31.6%) not treated with asfotase alfa due to its unavailability. Meanwhile, thirteen (68.4%) of our patients were treated with asfotase alfa for a median of 5.5 months [IQR: 3, 99], 9 (69.2%) were compliant, and 10 (76.9%) experienced a beneficial response. Patients demonstrated beneficial responses, including radiographic improvements, enhanced mobility, and reduced pain. Asfotase alfa resulted in a significant increase in the ALP levels from 24.5 [IQR: 12, 33.5] to 2,176 [IQR: 1,433, 6,000] U/L (P = 0.002), height from 66 [IQR: 56, 74] to 122 [IQR: 60, 123.5] cm (P = 0.018). The height SDS slightly improved from −4.69 [IQR: −6.51, −2.10] to −3.69 [IQR: −6.04, −2.47], but not statistically significant (P = 0.237). Furthermore, three (15.8%) patients received growth hormone. Complications related to the disease were observed in 11 patients (57.9%), including Craniosynostosis repair and seizures, and 4 (21.05%) patients died, all with perinatal-onset disease, and only one of them had received asfotase alfa, administered for just 3 months (Tables 3, 4).

**TABLE 3:** Treatment outcomes and complications related to the disease among patients (N = 19).

Variables	n (%), median {IQR}
Treatment	
Asfotase alfa	
Yes	13 (68.4)
No	6 (31.6)
Duration in months	5.5 {3.00, 99.00}
Response to asfotase alfa (n = 13)	
Beneficial	10 (76.9)
Initial benefit then regressed (was not compliant)	1 (7.7)
No change (perinatal form, short duration 1–2 months)	2 (15.4)
Growth hormone	
Yes	3 (15.8)
No	16 (84.2)
Compliant to asfotase alfa (n = 13)	
Yes	9 (69.2)
No	4 (30.8)
Adverse related to asfotase alfa (n = 13)	
Yes	1 (7.7)
No	12 (92.3)
Complications related to the disease	
Complications related to the disease (surgeries, infections)	
Yes	11 (57.9)
No	8 (42.1)
Specific complications (n = 11)^a^	
Craniosynostosis repair	5 (45.5)
Scoliosis repair	2 (18.2)
Frequent infection	2 (18.2)
Ventriculo-peritoneal shunt insertion	1 (9.1)
Seizures	4 (9.1)
Encephalomalacia	1 (9.1)
Respiratory failure	1 (9.1)
Died^b^	
Yes	4 (21.05)
No	15 (78.94)

^a^ 3 patients had more than one complication.

^b^ Three deaths in the perinatal group occurred at ages less than 4 months due to sepsis, and the remaining dead case was in the infantile group and died due to acute respiratory failure.

**TABLE 4:** Differences in height and laboratory findings of patients before and after asfotase alfa (N = 13).

Variables	Baseline median [IQR]	Last visit median (IQR)	P-value^a^
Height (cm) (n = 7)	66 [56, 74]	122 [60, 123.5]	0.018*
Height SDS (n = 7)	−4.69 [-6.51, −2.10]	−3.69 [-6.04, −2.47]	0.237
ALP (U/L) (n = 12)	24.50 [12, 33.50]	2,176 [1,433, 6,000]	0.002*
Blood Ca (mmol/L) (n = 12)	2.48 [2.43, 2.54]	2.49 [2.43, 2.58]	0.814
Phosphate (mmol/L) (n = 12)	1.77 [1.49, 2.19]	1.97 [1.59, 2.11]	0.784
Vitamin D (nmol/L) (n = 9)	77.37 [23.5, 94.5]	43 [20.5, 77.75]	0.314

^a^ Wilcoxon signed-rank test. *Statistical significance at P < 0.05.

SDS: standard deviation score.

Among the treated patients, three showed no therapeutic response. One patient initially responded but later experienced a regression. This patient was non-compliant with therapy, and treatment was started after the age of four. He had significant skeletal deformities. Two patients showed no change in their treatment response. Both initiated the therapy in the neonatal period—one at 2 weeks of age and the other at 3 months of age. The first patient was treated for a short duration of 2 months but ultimately passed away due to illness and sepsis. The second patient received treatment for only 1 month before also succumbing to sepsis. In both cases, early mortality and limited treatment duration likely contributed to the lack of therapeutic response (Table 3).

**FIGURE 2:**
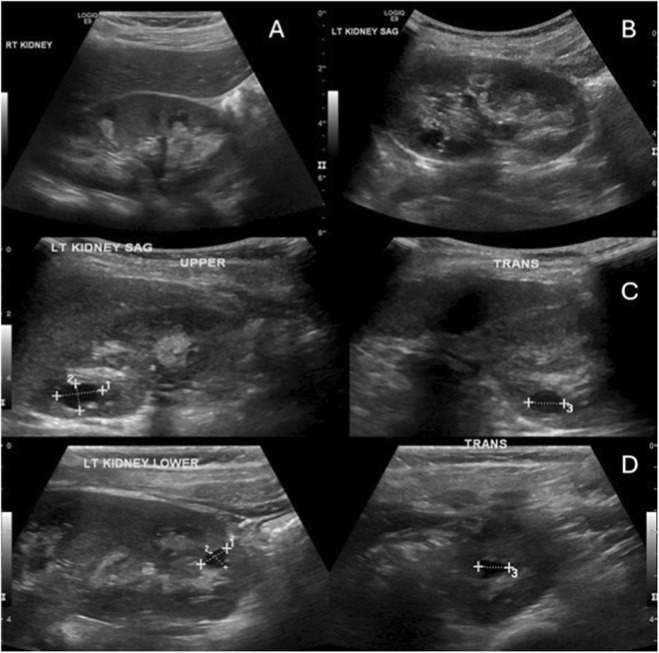
Renal ultrasound findings in an infantile female with hypophosphatasia (Case 12). The ultrasound reveals grade 2–3 medullary nephrocalcinosis and small simple cysts in the left kidney’s upper and lower poles. Sagittal views **(A,B)** show mildly increased parenchymal echogenicity with nephrocalcinosis in both kidneys. Additionally, sagittal **(C)** and transverse **(D)** images of the left kidney highlight a well-defined upper pole cyst (12 b 8 b 9 mm) and a smaller lower pole cyst (7 b 5 b 7 mm). The patient carries a homozygous ALPL gene mutation (NM_001369805.2:c.293C>T, p. (Ser98Phe)), classified as a variant of uncertain significance (VUS).

**FIGURE 3:**
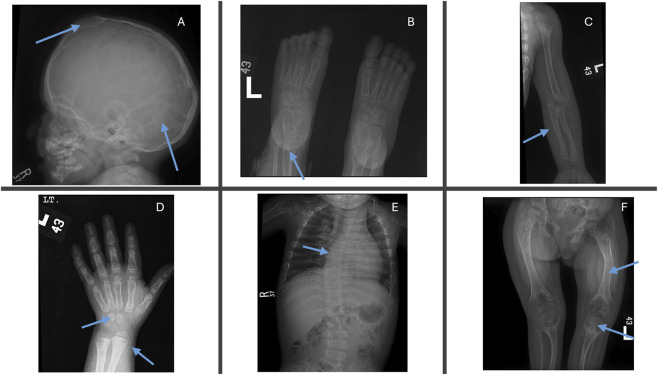
Skeletal survey at 1 year of age in an infantile hypophosphatasia case (Case 12). **(A)** Skull radiograph showing scalloping of the inner table cortices, bulging of the anterior fontanelle, and early fusion of the cranial sutures. **(B)** Foot radiograph demonstrating multiple bony lucencies and widened metaphyses, indicative of defective mineralization. **(C)** Upper limb radiograph showing cortical thinning and bowing of the long bones. **(D)** Hand radiograph revealing multiple bony lucencies, broadening of the metaphyses, and delayed ossification centers. **(E)** Spinal radiograph displaying thoracic kyphoscoliosis with generalized osteopenia. **(F)** Pelvic and lower limb radiograph illustrating bowing of the femurs, widened metaphyses, and poor mineralization of the epiphyses.

**FIGURE 4:**
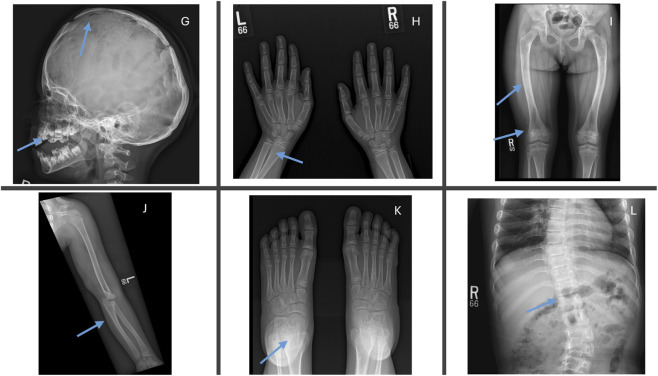
Follow-up skeletal survey at 6 years of age post-asfotase alfa therapy (Case 12). **(G)** Skull radiograph showing persistent lacunar appearance, mild midface hypoplasia, and abnormal dentition. **(H)** Hand radiograph demonstrating improved mineralization but persistent cortical thinning and mild negative ulnar variance. **(I)** Lower limb radiograph showing persistent bowing of the femurs and improvement in metaphyseal morphology. **(J)** Upper limb radiograph depicting mild negative ulnar variance bilaterally. **(K)** Foot radiograph with evidence of broadening and widening of the tarsal and metatarsal bones. **(L)** Spinal radiograph revealing an S-shaped thoracolumbar scoliosis with focal kyphosis at the thoracolumbar junction.

We comprehensively investigated the radiological changes of an infantile case (Case 12) who was responsive to asfotase alfa to evaluate skeletal and renal changes over time. The initial renal ultrasound (Figure 2) revealed grade 2–3 medullary nephrocalcinosis and small simple cysts in the left kidney’s upper and lower poles. The baseline skeletal survey at 1 year of age (Figure 3) demonstrated severe mineralization defects, including cranial suture abnormalities, metaphyseal widening, cortical thinning, and thoracic kyphoscoliosis. A follow-up skeletal survey at 6 years post-asfotase alfa therapy (Figure 4) showed improvement in mineralization; however, persistent skeletal abnormalities remained, including femoral bowing, spinal scoliosis, and cortical thinning, underscoring the long-term skeletal consequences of HPP despite enzyme replacement therapy.

**FIGURE 5:**
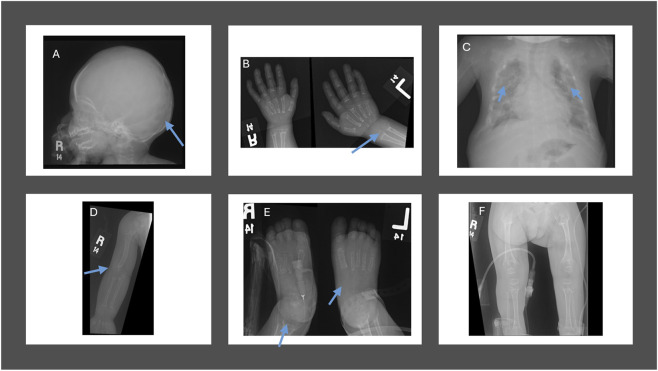
Skeletal survey at 1 years of age in an infantile hypophosphatasia case (Case 15). **(A)** Skull x-ray showed poor ossification of the calvarium, especially the occipital and parietal bone, with generalized osteopenia. **(B)** Hand radiograph revealing multiple bony lucencies, broadening of the metaphysis, and delayed ossification centers. **(C)** Thin osteopenic ribs are noted, associated with bilateral multiple fractures. **(D)** Cannot distinguish the margins of the metaphysis, demonstrating multiple bony lucencies and widened metaphysis, indicative of defective mineralization. **(E)** Osteopenia in bilateral feet with fraying of the distal tibia and fibula. **(F)** Increase in distance of the metaphyseal ends. Metaphyseal flaring of the knees. Convexity of the distal metaphysis of the femur. Faint periosteal reactions in the femur.

**FIGURE 6:**
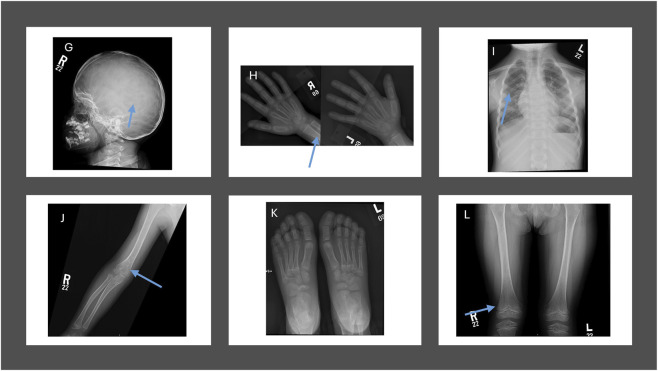
Follow-up skeletal survey at 6 years of age post-asfotase alfa therapy (Case 15) Generalized improvement in bone mineralization. **(G)** All the sutures are fused suggestive of craniosynostosis. Indentation on the inner table of the calvarium from the growth of the brain. **(H)** The distal radius and ulna appear well-formed, and the metaphyseal and epiphyseal regions are unremarkable. **(I)** No visible fractures or destructive bone lesions. **(J)** Distance of the metaphysis improved along with the shape. Well defined margins of the distal humerus. **(K)** The bones of both feet appear well-formed, symmetric, with no evidence of deformity or fracture. **(L)** Sclerosis of the metaphysis of the femur which indicates treatment response.

Additionally, the skeletal survey of Case 15 at 1 year of age (Figure 5) showed poor ossification of the calvarium, generalized osteopenia, multiple bony lucencies, broadening of the metaphysis, delayed ossification centers, thin osteopenic ribs with bilateral multiple fractures, and metaphyseal flaring. The follow-up survey at 6 years post-asfotase alfa therapy (Figure 6) showed generalized improvement in bone mineralization, with well-formed bones, no evidence of deformity or fracture, and sclerosis of the metaphysis of the femur indicating treatment response.

### *ALPL* genotype among the study population

Genetic testing was performed using exome sequencing or *ALPL* NGS in 19 individual cases affected with Hypophosphatasia from Saudi Arabia (Table 5). A molecular genetic diagnosis was established in the families studied. A total of six different variants were identified in the study, where five were missense mutations and one was non-coding. All detected missense variants were classified as pathogenic variants according to the ACMG classification, and the non-coding variant NM_000478.6:c.793–3T>G was classified as a variant of uncertain significance. All detected variants in our cohort were in the homozygous state.

**TABLE 5:** Autosomal recessive genetic variants in the ALPL gene among 19 patients.

Nucleotide change (MANE transcript)	Exon	Amino acid change	ACMG classification	Reported before	Mutation type	Gender n (%)	Disease onset	Region	Died	Total (%)
NM_000478.6: c.214A>G	4	p. (Ile72Val)	Pathogenic	Yes	Missense	Male 2 (50) Female 2 (50)	3 infantile	3 AlAhsa	No	4 (21%)
							1 childhood	1 Riyadh		
NM_000478.6: c.293C>T	4	p. (Ser98Phe)	Pathogenic	No	Missense	Male 3 (60) Female 2 (40)	4 infantile	4 Riyadh	No	7 (37%)
							1 perinatal	1 Madinah		
NM_000478.6: c.340G>A	5	p. (Ala114Thr)	Pathogenic	Yes	Missense	Male 1 (100)	Perinatal	Riyadh	Yes	1 (5.3%)
NM_000478.6: c.793–3T>G	Intron 7	NA	VUS	No	Splicing	Male 1 (100)	Perinatal	Riyadh	Yes	1 (5.3%)
NM_000478.6: c.815G>A	8	p. (Arg272His)	Pathogenic	Yes	Missense	Male 1 (100)	Infantile	Riyadh	No	1 (5.3%)
NM_000478.6: c.977G>T	9	p. (Gly326Val)	Pathogenic	No	Missense	Male 3 (60) Female 2 (40)	3 infantile	2 Taif	2 Yes	5 (26%)
							2 perinatal	2 Riyadh	3 No	
							​	1 Eastern	​	

The most common variant detected in the cohort was the novel missense variant c.293C>T (p.Ser98Phe) that was found in seven cases, which represented almost 37% (7/19) of the patients and was linked to both infantile and perinatal onset HPP.

The novel missense variant c.977G>T (p.Gly326Val) was found in 5 patients of the cohort (26%). The variant was associated with infantile and perinatal onset HPP.

According to the ACMG classification, both variants c.293C>T (p.Ser98Phe) and c.977G>T (p.Gly326Val) should be classified as pathogenic because they were observed in 7 and 5 families, respectively.

The previously reported pathogenic variant c.214A>G (p.Ile72Val) was observed in 4 families and was associated with infantile and childhood-onset HPP. The pathogenic missense variant c.815G>A (p.Arg272His) was observed in one case with infantile onset. The known pathogenic variant (p.Ala114Thr) was detected in the mother of the HPP case with perinatal onset, who passed away before the blood sample could be collected. Both parents were heterozygous for the variant, and the deceased son was presumed to be homozygous. The novel non-coding variant c.793 3T>G was detected in one case with perinatal onset. The variant is expected to abolish splicing and lead to the loss of function of the enzyme. According to the ACMG classification, the variant was classified as a variant of uncertain significance (VUS; PM2, PP3, PP4). Aggregated *in silico* prediction tools predicted the pathogenic support.

## Discussion

HPP is a rare genetic metabolic disease with a variable clinical spectrum of presentation and complications. Until now, there have been few HPP case reports in Saudi Arabia. Here, we described the demographic, clinical, and genetic features of 19 HPP cases from 6 medical centers across Saudi Arabia.

We observed male predominance among our cohort with a male-to-female ratio of 13:6. According to previous reports, the gender distribution of HPP tends toward male predominance among children and female predominance among adults (Whyte et al., 2015; Weider et al., 2022; Li et al., 2023; Liu et al., 2021; Glotov et al., 2022). However, Martos-Moreno et al. observed a balanced sex distribution of HPP among children based on data from the Global HPP Registry (Martos-Moreno et al., 2023). Vogt and his colleagues also reported a similar gender distribution (Vogt et al., 2020).

Furthermore, the perinatal lethal type of HPP was more frequent among males than females (Taketani et al., 2014; Liu et al., 2021). These findings highlight the necessity of investigating the potential association between genetic predispositions and gender. Additionally, there was a high prevalence of consanguinity and positive family history among the parents of our population, which suggests that consanguinity may increase the likelihood of inheritance of HPP, specifically the autosomal recessive type, due to shared alleles, and highlights the importance of early genetic screening and counselling of consanguineous couples (Gor et al., 2000; Khayat et al., 2024).

We found that the most common form of HPP in our cohort was infantile HPP, followed by perinatal and childhood HPP. The clinical heterogeneity of HPP with overlapping signs and complications is evident in the existing literature. For instance, the perinatal lethal form was the most prevalent in Japanese children, and the childhood form was most frequent in Russian and German children (Liu et al., 2021; Michigami et al., 2020).

This variability in the clinical spectrum of HPP is significantly associated with diagnostic delay, emphasizing the need for timely genetic testing and diagnosis of suspected cases, raising awareness, and establishing a definitive diagnostic guideline (Vogt et al., 2020; Khan et al., 2024).

Bone deformities, including Genu varum or genu valgum Scoliosis, and various other bone presentations were observed among 100% of our cohort, mainly in the skull or limbs. Other common manifestations included craniosynostosis (78.5%), fractures (42.1%), nephrocalcinosis (26.3%), and kyphoscoliosis (49.9%). Besides, craniosynostosis repair and scoliosis repair were performed for five and two patients, respectively. These findings align with those documented in other studies (Glotov et al., 2022; Högler et al., 2019; Szabo et al., 2019). Furthermore, failure to thrive, premature tooth loss, gross motor/ambulation difficulties, and musculoskeletal pain are among the most common manifestations of pediatric HPP patients (Vogt et al., 2020; Whyte et al., 2016a). Therefore, early recognition of different systemic manifestations of HPP in suspicious cases is crucial to ensure a timely diagnosis.

Bone manifestations are hypothesized to result from hypomineralization due to pyrophosphate (PPi) accumulation, which occurs because of insufficient TNSALP activity in osteoblasts. Additionally, infantile HPP has been associated with the premature closure of cranial sutures, leading to craniosynostosis (Montero-Lopez et al., 2024). Besides, bone hypomineralization can cause hypercalcemia and hypercalciuria, potentially leading to nephrocalcinosis and decreased parathyroid hormone levels (Bacchetta, 2017).

Although the mechanism behind the neurological symptoms in HPP remains unclear, convulsions were noted in 26.3% of our cohort. This is thought to result from vitamin B6 deficiency in the central nervous system. Colazo et al. observed a high prevalence of neurological symptoms among HPP patients compared to the general population, including fatigue, headache, sleep disturbance, gait change, vertigo, depression, anxiety, neuropathy, and hearing loss (Colazo et al., 2019). These symptoms are common yet often unnoticed, underscoring the need for increased awareness to ensure timely diagnosis and detection of the disease.

Genetic analysis is of value in confirming the diagnosis of HPP. Our analysis revealed several mutations in *ALPL*. The most common variants, c.293C>T (p.Ser98Phe) and c.977G>T (p.Gly326Val), were found in 37% and 26% of patients, respectively, and were associated with both infantile and perinatal onset HPP. The c.293C>T (p.Ser98Phe) and c.977G>T (p.Gly326Val) variants in the *ALPL* gene have not been previously reported in major genetic databases such as ClinVar or gnomAD, suggesting that they are novel mutations. Given their recurrence in multiple unrelated individuals from the same geographic or ethnic group, these mutations may represent founder mutations.

Consistent with the literature, the detected mutations were missense and classified as pathogenic variants. There is scarce data regarding the genetic landscape of HPP in Saudi Arabia and the Middle East. A prior case report by Alotaibi and Amal identified a novel homozygous c.173T>C transition in exon 3 of the *ALPL* gene in a Saudi infant with infantile HPP (Alotaibi and Amal, 2017).

Compared with other regions, c.1559delT, p. Asp378Val, and p. Glu191Lys were the most common mutations in Japan (Michigami et al., 2020), United States (Whyte et al., 2015), and Europe (Mornet et al., 2020), respectively. Similar to our observation, Mornet et al. also pointed out that the alleles detected in the Middle East were primarily recessive, possibly due to the high prevalence of consanguinity, which results in a high number of homozygotes (Mornet et al., 2020). These results indicated that a specific screening panel may be necessary for diagnosing Saudi HPP patients.

According to our clinical observations, c.977G>T (p.Gly326Val) was detected among five patients, and two of them died. The variant was associated with severe HPP symptoms, including bowing of the lower extremities, micromelia, bone spurs, small thoracic cage, fragment skull bone, osteopenia, metaphyseal changes, and neurological manifestations such as convulsions, meningoencephalitis, and encephalomalacia. Meanwhile, patients with the c.293C>T (p.Ser98Phe) variant usually suffered from craniosynostosis and bone deformities.

Previous studies have reported that the broad spectrum of *ALPL* mutations was linked to high phenotypic variability among HPP patients. This suggests that additional genetic, epigenetic, and environmental factors might be involved in the genotype-phenotype association (Hofmann et al., 2014; Mornet et al., 2013).

Moreover, variants of uncertain significance (VUS) among our population in general may also pose challenges to the diagnosis and the treatment of the patients, and prolong diagnostic delay (Farman et al., 2024). Functional studies are necessary to elucidate the pathogenicity of these variants and improve the accuracy of genetic counseling.

Enzyme replacement therapy (ERT) has emerged as a promising treatment for patients with pediatric-onset HPP (Jaswanthi et al., 2023). Asfotase alfa s a tissue-nonspecific ALP enzyme ERT which has be shown to improve various skeletal and non-skeletal manifestations in both pediatric and adult patients with an acceptable safety profile and manageable side effects (Khan et al., 2025). In our cohort, thirteen patients received asfotase alfa; ten responded positively, while two showed no change due to early mortality and limited treatment duration, and one initially responded but later experienced regression, redness, and an injection site reaction due to lack of compliance. Moreover, 84.6% (11 out of 13) of treated patients were alive at the end of the study.

In a previous 7-year follow-up clinical trial, asfotase alfa resulted in sustained improvements in skeletal mineralization, respiratory function, growth, and cognitive and motor function (Whyte et al., 2019). Shirinezhad et al. have highlighted that asfotase alfa–related adverse events were generally mild and commonly included injection site reactions and pyrexia (Shirinezhad et al., 2024). Furthermore, Whyte et al. demonstrated that asfotase alfa significantly improved survival rates among perinatal and infantile HPP patients (Whyte et al., 2016b).

At baseline, we observed a significant reduction in serum ALP levels among HPP patients. Following treatment with asfotase alfa, serum ALP levels sharply rose (P = 0.002). This is consistent with the findings of Hofmann et al., who reported a sustained elevation of ALP levels, as expected with asfotase alfa treatment (Hofmann et al., 2019). These findings underscore the potential benefits of asfotase alfa and its excellent safety profile.

Thus, HPP diagnosis remains challenging due to phenotypic variability and a lack of awareness. Diagnostic delays are common worldwide, emphasizing the need for improved screening programs.

Our study has several limitations, including the retrospective design and small sample size, which may limit the generalizability and interpretation of our findings. Also, not all clinical or biochemical data were available in the medical records, resulting in incomplete data capture and recall bias.

## Conclusion

This study provides novel insights into the clinical and genetic landscape of HPP in Saudi Arabia. Infantile-onset HPP predominates, with distinct *ALPL* mutations compared to Western populations. Asfotase alfa demonstrated strong therapeutic benefits, but response variability warrants further research into genetic predictors, as well as a closer examination of patients’ compliance with treatment. The burden of the disease, reflected in the increased incidence of bone and neurological manifestations, determine that it is necessary to implement strategies for an earlier diagnosis that allows to achieve adequate management. Thus, a Saudi-specific genetic screening panel could enhance early detection, given the unique *ALPL* variants identified in this study. The high consanguinity rate necessitates preconception and prenatal genetic counselling to support informed reproductive decision-making and reduce severe HPP incidence. The detection of novel variants among HPP patients expands our knowledge of genetic landscape of the disease and reinforces the importance of population-specific molecular diagnostics. Future Research should be conducted in a larger cohort to strengthen genotype-phenotype correlations. Additionally, functional studies of *ALPL* variants should assess the impact on enzyme activity, and longitudinal follow-up should evaluate the long-term outcomes of enzyme replacement therapy. Also, a multidisciplinary management of HPP in high-risk, high-consanguinity settings such as Saudi Arabia is warranted.

## Data Availability

The original contributions presented in the study are included in the article/supplementary material, further inquiries can be directed to the corresponding authors.
